# Nurses’ knowledge and understanding of obstacles encountered them when administering resuscitation medications: a cross-sectional study from Palestine

**DOI:** 10.1186/s12912-022-00895-1

**Published:** 2022-05-16

**Authors:** Rawan I. Qedan, Marah A. Daibes, Samah W. Al-Jabi, Amer A. Koni, Sa’ed H. Zyoud

**Affiliations:** 1 Department of Clinical and Community Pharmacy, College of Medicine and Health Sciences, An-Najah National University, Nablus, 44839 Palestine; 2 Division of Clinical Pharmacy, Department of Hematology and Oncology, An-Najah National University Hospital, Nablus, 44839 Palestine; 3 Poison Control and Drug Information Center (PCDIC), College of Medicine and Health Sciences, An-Najah National University, Nablus, 44839 Palestine; 4 Clinical Research Center, An-Najah National University Hospital, Nablus, 44839 Palestine

**Keywords:** Knowledge, Resuscitation medications, Medication errors, Nurses, Palestine, Cross-sectional

## Abstract

**Background:**

Medication errors (ME) are one of the most important reasons for patient morbidity and mortality, but insufficient drug knowledge among nurses is considered a major factor in drug administration errors. Furthermore, the complex and stressful systems surrounding resuscitation events increase nursing errors.

**Aims:**

This study aimed to assess the knowledge about resuscitation medications and understand the obstacles faced by nurses when giving resuscitation medications. Additionally, errors in the reporting of resuscitation medication administration and the reasons that prevented nurses from reporting errors were investigated.

**Methods:**

A cross-sectional study was conducted in the West Bank, Palestine. Convenient sampling was used to collect data, which was collected via a face-to-face interview questionnaire taken from a previous study. The questionnaire consisted of five parts: demographic data, knowledge of resuscitation medications (20 true/false questions), self-evaluation and causes behind not reporting ME, with suggestions to decrease ME.

**Results:**

A total of 200 nurses participated in the study. Nurses were found to have insufficient knowledge about resuscitation medications (58.6%). A high knowledge score was associated with male nurses, those working in the general ward, the cardiac care unit (CCU), the intensive care unit (ICU) and the general ward. The main obstacles nurses faced when administering resuscitation medication were the chaotic environment in cardiopulmonary resuscitation (62%), the unavailability of pharmacists for a whole day (61%), and different medications that look alike in the packaging (61%). Most nurses (70.5%) hoped to gain additional training. In our study, we found no compatibility in the definition of ME between nurses and hospitals (43.5%).

**Conclusions:**

Nurses had insufficient knowledge of resuscitation medications. One of the obstacles nurses faced was that pharmacists should appropriately arrange medications, and nurses wanted continuous learning and additional training about resuscitation medications to decrease ME.

## Background

One of the most important reasons for patient morbidity and mortality is medication errors (ME) [1]; furthermore, it is thought to be the most important cause of preventable harm to patients [2]. ME are a dangerous problem during treatment, especially for patients in intensive care units (ICUs) who can suffer very serious complications due to their severe illnesses and the complex pharmacotherapy programs they receive [3].

Any errors in dose, administration rate, drug concentration, type of drug, route of administration, method of administration or delay in administration, fall under the definition of ‘ME’ [4]. Research has found that there can be important errors during the preparation and administration of medications [4–6]. There are many causes of EM, and many research studies have focused on these reasons; their findings include stressful and complex systems, workload, poor skill, distracting nurses during their work, lack of concentration, and insufficient knowledge of drug and calculation tasks, confusion between similar and sound-alike medications, use of medications with a narrow therapeutic index (NTI), and insufficient experience in nurses’ work [4, 5, 7–10]. Furthermore, other research has found that serious incidents occur due to problems related to teamwork and knowledge related to cardiac arrest in special cases [11].

In general, the prevalence of ME was 32.1% [12] to 94% [13]. Importantly, approximately 38% of ME are caused by nurses [14]. The prevalence of ME among hospital nurses was 17% [15], while it was 39.68% among nursing students [16], and both percentages are considered high. Furthermore, a systematic review found a prevalence between 16–27% [17]. In countries in the Middle East, studies related to ME are few and do not contain sufficient information [18]. A Jordanian analysis demonstrated that the size of the hospital may play a role in the nursing rate of reporting ME and their views on this regard [19]. In the same country, lack of experience and knowledge, along with workload, was documented as the main factors behind medication error [20]. Furthermore, a retrospective study in a teaching hospital showed that 14.3% of incidents are related to ME [21]. In another Middle East country, Saudi Arabia, medication errors were found to be high in a hospital during the COVID-19 crisis as 19% [22]. Researchers explain that due to work overload among healthcare providers [22].

Unfortunately, many hospitals do not have a special form or internal system for reporting errors [23], and sometimes, health care professionals tend to report errors voluntarily and using verbal methods instead of a written or documented report [23, 24]. This led to underreporting of ME, which are estimated to be 50–60% each year [23]. Different ways of reporting errors were identified, including verbal and written on a paper [25], and the most recent one is web-based forms [26]. The consequences of ME on healthcare professionals include providing further training, improving communication, and informing the personnel who committed the error [27].

Resuscitation is a multipart process executed in a short time, frequently occurring in uncontrolled situations; a stressful situation that requires nurses with high knowledge, and where, with limited information and time nurses, should respond as fast as possible. In such conditions, nurses' performance may not be satisfactory enough, particularly when nurses are not qualified or experienced enough to respond to stressful conditions [11, 28]. Confidentiality in the resuscitation environment can generate errors in the critical stages of medication administration [29].

Nurses lacking knowledge of drugs and their appropriate doses can cause medication errors [3, 30], especially with increased stress and interruptions during medication administration among nurses [31]. The resuscitation procedure requires a rapid response of nurses to the doctor’s oral orders without enough time to get reference information on administering a resuscitation medication [32].

In addition, previous research worldwide has produced few studies on nurses' knowledge of resuscitation medications and their obstacles during medication administration. This study is considered to be the first in Palestine. To reduce morbidity and mortality among patients with ME [33], it is necessary to assess the pharmacological knowledge and to understand the obstacles they face when administering resuscitation medications. Nursing associations can then argue for mandatory training courses on administering resuscitation medications and decreasing the obstacles they encounter to minimise resuscitation ME as much as possible. This research will help university academics design multidisciplinary courses in clinical pharmacology focusing on resuscitation medication as part of ongoing nurse education that meets the needs of Palestinian nursing practice situations.

The administration of medication during resuscitation must be performed correctly, as incorrect or delayed drug administration can result in serious harm or death to patients. Therefore, the study objectives were: 1) to assess nurses’ knowledge about resuscitation medications, 2) to determine the obstacles that nurses face when giving resuscitation medications, 3) to evaluate resuscitation medication administration errors in reporting and the reason that prevent nurses to not report the errors, and 4) to determine factors that affect sufficient knowledge among nurses.

## Methods

### Study design

We used a cross-sectional design, which is an observational method applied to assess knowledge among health members at a specific point in time [34, 35]. In fact, we evaluated the knowledge and understanding of nurses about the obstacles they face when administering resuscitation medications.

### Study setting

Palestine consists of two zones: the West Bank and the Gaza Strip, with a total population of about five million inhabitants. Nearly 60.2% live in the West Bank and 39.8% live in the Gaza Strip. The West Bank is divided into three regions and 11 governorates. The north area comprises: Jenin, Tulkarm, Nablus, Qalqilya and Tubas; the middle area comprises: Jerusalem, Ramallah, Salfit, and Jericho; the south area comprises: Bethlehem and Hebron [36].

This study was carried out in the north district of the West Bank of Palestine, where a list of hospitals and their addresses was acquired from the Ministry of Health. Based on the lists, the study held the following governmental hospitals in the north of the West Bank: Nablus, Jenin, Tulkarm, Qalqilya, and Tubas.

### Study population

The population was chosen from nurses who worked in governmental hospitals in the north of the West Bank. 4362 registered nurses work in governmental health care units in Palestine. There are seven universities in the West Bank from which nurses with different specialties graduate [36].

### Sampling procedure and sample size calculation

This study used convenience samples from nurses from government hospitals in the north district of the West Bank of Palestine, from May 2019 to February 2020. We interviewed all study participants in the wards of the above mentioned hospitals. First, all aspects of the study were explained in detail. Second, informed verbal consent was obtained. Third, we collected all data, including sociodemographic data from the participants themselves through a face-to-face interview. Each interview took about 15 min.

The data from the Palestinian Health Information Centre in 2014 found that 1566 nurses worked in the governmental hospitals in the West Bank of Palestine [37]. Generally, we assumed that about 400 nurses who worked in hospitals would be incorporated in the current study. We calculate the sample size for our study using the Raosoft sample size calculator (http://www.raosoft.com/samplesize.html). The sample size was 200 nurses, to achieve a confidence level of 95% and a margin of error of 5%.

### Inclusion and exclusion criteria

The inclusion criteria were nurses of Palestinian nationality and licensed by the Palestinian Ministry of Health; having at least diploma or higher degree; and were working in ER, ICU, MW or paediatric departments. Exclusion criteria were nurses who refused to participate in the study, students from nursing school, and those who worked in private or teaching hospitals.

### Instruments and data collection form

The questionnaire used consisted of five parts that had been developed by the previous studies [32, 38–42]. The prepared questionnaire consisted of open and closed questions. The questionnaire contained five parts:

- The first part was about demographic data, which contained questions about age, gender, the region of residence, marital status, educational level, position, years of work experience, CPR experience, and training that can affect knowledge of resuscitation medications.
- In the second section, we evaluated nurses' knowledge of resuscitation medications, which consisted of 20 questions. The degree of knowledge about resuscitation medications was determined according to the nurse’s score. From the choices of true/false/I don’t know, we calculated the correct answer rate on knowledge of pharmacology and analyzed the effects of demographic data on knowledge score.
- The third section was designed for nurse self-evaluation for the following three factors regarding resuscitation medications:
  - Obstacles they faced and reasons for why medication administration errors occurred, indicating their level of agreement using a five-point Likert-type scale with fixed values ranging from 5 = ‘strongly agree’ to 1 = ‘strongly disagree’ for 20 items.
  - The degree of their level (five levels to choose from ‘sufficient’ to ‘extremely insufficient’).
  - Their need for training (three choices: ‘need’, ‘no comment’, and ‘no need’).
- The fourth section included 15 items to find the causes behind not reporting ME. Nurses were asked to indicate their level of agreement using a five-point Likert-type scale with fixed values ranging from 5 = ‘strongly agree’ to 1 = ‘strongly disagree’.
- The fifth part consisted of five items regarding suggestions to decrease ME. Nurses were asked to choose their level of agreement using a five-point Likert scale with fixed values ranging from 5 = ‘strongly agree’ to 1 = ‘strongly disagree’.

### Ethical approval

All aspects of the study protocol, including access to and use of the information of the participants, were approved by the *Institutional Review Boards of An-Najah National University* (IRB) (Ethical approval code: #7 May 2018). Furthermore, we obtained approval from the local health authorities of the four hospitals studied. Verbal consent was obtained from all participants.

### Pilot study

The validity of the questionnaire's content was checked by consensus of a group of three experts in the field drawn from academia (two experts in clinical pharmacy and one expert in clinical pharmacology). All experts confirmed that the questionnaire issues strictly adhered to the goals of the research. A pilot study (25 participants) was conducted to ensure the simplicity of the availability of the study questions, ensure the required data, estimate the time required, and modify the data collection form as appropriate. Nurses who participated in the pilot study were excluded from the final analysis.

### Statistical analysis

Data were entered and analyzed using the Statistical Package for Social Sciences programme version 15 (SPSS). We expressed the data as continuous means ± standard deviation (SD) variables and as frequencies (percentages) for categorical variables. Non-normally distributed variables were expressed as medians (lower–upper quartiles). The normality of the variables was tested using the Kolmogorov–Smirnov test. The chi-square or the Fisher exact test was used to test significance between categorical variables, as appropriate. We used the Kruskal–Wallis test or Mann–Whitney U test to test for differences in the mean between categories. The significance level was established at *p* < 0.05.

## Results

### Sociodemographic data

This study was hospital-based in health care with a cross-section method, which was carried out with 200 nurses who worked in government hospitals in the north of West Bank of Palestine. As Table 1 indicates, half of the participants (approximately 51.5%) were women. Most of them (82%) were younger than 40 years of age and most of the participants were married (81.5%). Other demographic characteristics of nurses are represented in Table 1.

**Table 1:** Socio-demographic characteristics of the study sample

Variable	Frequency (%) N = 200
**Gender**	
Male	97 (48.5)
Female	103 (51.5)
**Age category (Years)**	
20–29	72 (36.0)
30–39	93 (46.5)
40–49	28 (14.0)
50–59	7 (3.5)
**Marital status**	
Single, divorced, widow	37 (18.5)
Married	163 (81.5)
**Hospital**	
Rafedia	35 (17.5)
Al-watany	33 (16.5)
Jenin	27 (13.5)
Tubas	21 (10.5)
Tulkarm	45 (22.5)
Qalqelia	39 (19.5)
**Department**	
ER	35 (17.5)
ICU	27 (13.5)
NICU	21 (10.5)
Paediatric	33 (16.5)
Men ward	41 (20.5)
Women Ward	28 (14.0)
CCU	7 (3.5)
Gynaecological ward	4 (2.0)
General	4 (2.0)
**Position**	
Staff nurse	185 (92.5)
Head nurse	11 (5.5)
Supervisor	4 (2.0)
**Residency**	
Tulkarm	47 (23.5)
Nablus	67 (33.5)
Jenin	37 (18.5)
Qalqelia	40 (20.0)
Tubas	9 (4.5)
**Years of working**	
Less than 5 years	49 (24.5)
5 to less than 10	71 (35.5)
10 years or more	80 (40.0)
**Educational status**	
Diploma	51 (25.5)
BS	129 (64.5)
MS	20 (10.0)
**CPR Training**	
Yes	175 (87.5)
No	25 (12.5)

*ER* emergency room, *ICU* intensive care unit, *NICU* neonatal intensive care unit, *MW* medical word, *CCU* cardiac/coronary care unit, *BS* bachelor degree, *MS* master degree

### Knowledge about the administration of resuscitation medications

We asked nurses about the proper administration of resuscitation medications, and the correct response rate was 58.6%; 28.2% were incorrect answers, and 13.2% answered ‘don’t know’. We found that question number 11, about the use of atropine in the treatment of pulseless electrical activity, received the lowest correct response rate: only 36.5% answered correctly. On the contrary, the highest correct response rate was 87.5%, agreeing that KCl is not administered as a fast IV push in an emergency event such as ventricular fibrillation. More than half of the nurses did not understand that when they calculated the dose of epinephrine (adrenaline) for children, they must use body weight and not body surface area (correct rate 44.5%). Furthermore, most nurses thought that drugs should be available in multiple concentrations to choose, although this is unacceptable (correct rate 41.5) (Table 2).

**Table 2:** Resuscitation medications administration knowledge

Questions	Answer	Correct answer	Incorrect answer	Don’t know the answer
		**n (%)**	**n (%)**	**n (%)**
1. 10 mls fast IV push of 15% KCl is given in an emergency cases for example ventricular fibrillation case	F	175 (87.5)	13 (6.5)	12 (6.0)
2. In the cardiac arrest case we give rapid IV push 1 mg epinephrine within 3–5 min	T	138 (69)	46 (23.0)	16 (8.0)
3. We favoured small venous vessels in case of dopamine injection	F	139 (69.5)	37 (18.5)	24 (12.0)
4.To preserve norepinephrine bitartrate effect we add glucose water to it	T	107 (53.5)	64 (32.0)	29 (14.5)
5. We inject NaHCO_3_ with epinephrine to cause an additive effect of the drug	F	115 (57.5)	32 (16)	53 (26.5)
6. To avoid hypoglycaemia occurrence, glucose water should constantly be given when starting CPR procedure	F	112 (56.0)	64 (32.0)	24 (12.0)
7. We can interchange between 10% Ca glucose and 10% CaCl_2_ because they are the same drug	F	136 (68.0)	20 (10.0)	44 (22.0)
8. Amiodarone is used to treat bradycardia	F	138 (69.0)	43 (21.0)	19 (9.5)
9. Nitroglycerine is used to treat cardiac infarction, which is accompanied by a drop in blood pressure and bradycardia	F	133 (66.5)	50 (25.0)	17 (8.5)
10. 1 amp of 1:1000 epinephrine is given as fast IV push in case of a mild allergic reaction	F	110 (55.0)	64 (32.0)	26 (13.0)
11. In the case of pulseless electrical activity, atropine is used within the treatment	F	73 (36.5)	112 (56.0)	15 (7.5)
12. Atracurium should be stored with other drugs and easily accessed by nurses	F	114 (57.0)	64 (32.0)	22 (11.0)
13. If we give the drugs endotracheally, the dosage should be increased 5 to 10 times than IV dose	F	103 (51.5)	48 (24.0)	49 (24.5)
14. To avoid bradycardia, give a small dose of atropine (< 0.5 mg) in case of CPR	F	101 (50.0)	77 (38.5)	22 (11.0)
15. Adenosine is given for bradycardia as slow IV drip (> 10 min)	F	130 (65.0)	54 (27.0)	16 (8.0)
16. The first choice of treatment in case of ventricular tachycardia or fibrillation is lidocaine	F	119 (59.5)	56 (28.0)	25 (12.5)
17. Rapid IV push 10% CaCl_2_10 ml over 1–2 min is given in the emergency cases	F	93 (46.5)	70 (35.0)	37 (18.5)
18. Various concentrations of all drugs should be available to choose by nurses	F	83 (41.5)	102 (51.0)	15 (7.5)
19. Epinephrine dose calculation is based on body surface area in paediatric CPR	F	89 (44.5)	89 (44.5)	22 (11.0)
20. Amiodarone is best given endotracheally, to increase its effect	F	137 (68.5)	25 (12.5)	38 (19.0)
Mean		58.6	28.2	13.2

*IV* intravenous, *KCl* potassium chloride, *NaHCo3* sodium bicarbonate, *CPR* Cardiopulmonary resuscitation, *Ca* calcium, *CaCl2* calcium chloride, *Amp* ampoule

### knowledge score and sociodemographic variables

The knowledge scale consisted of 20 questions to measure the knowledge among nurses about resuscitation medications. As shown in Table 3, the median knowledge score for male participants was high (13; quartile range 10–16) compared to females. The median knowledge for age category, 20 to 29 years was 12.5 (9–16), 30 to 39 years was 12 (8–15), 40–49 years was 11 (8.25–14.75), and 50 to 59 years was 13 (9–15), respectively. The participants who married had a median knowledge score of 12 (9–15), while the single, divorced and widowed nurses had a score of 13 (8.5–15). A high median knowledge score was observed in nurses who worked in the CCU department (16; 15–17), and that worked in the general wards 17.5 (14.75–18.75) and whose position was a supervisor 17.5 (14.75–18.75). Furthermore, the years of working showed the same median knowledge scale (12; 8–15.5); nurses having a master’s degree showed a higher knowledge score, 14 (9.25–15.75), and also those who had CPR training showed a higher knowledge score, 13 (9–15).

**Table 3:** Knowledge score by socio-demographic variables

Variable	Frequency (%)	Median k (Q1–Q3)	Mean rank	*P*- value
**Gender**				
Male	97 (48.5)	13 (10–16)	114.59	0.001^a^
Female	103 (51.5)	10 (8–14)	87.23	
**Age category (Years)**				
20–29	72 (36.0)	12.5 (9–16)	106.58	
30–39	93 (46.5)	12 (8–15)	98.77	
40–49	28 (14.0)	11 (8.25–14.75)	91.45	0.661^b^
50–59	7 (3.5)	13 (9–15)	97.14	
**Marital status**				
(single, divorced, widow)	37 (18.5)	13 (8.5–15)	100.82	
Married	163 (81.5)	12 (9–15)	100.43	0.970^a^
**Hospital**				
Rafedia	35 (17.5)	12 (8–14)	94.13	
Al–watany	33 (16.5)	13 (10–16)	112.74	
Jenin	27 (13.5)	11 (8–15)	96.39	0.194^b^
Tubas	21 (10.5)	11 (8–14)	92.95	
Tulkarm	45 (22.5)	14 (10–16)	114.59	
Qalqelia	39 (19.5)	10 (7–15)	86.51	
**Department**				
ER	35 (17.5)	13 (10–15)	109.46	
ICU	27 (13.5)	14 (11–15)	119.72	
NICU	21 (10.5)	12 (8–16)	99.02	
Paediatric	33 (16.5)	9 (7.5–13)	73.2	0.000^b^
Men Ward	41 (20.5)	11 (9–16)	101.78	
Women Ward	28 (14.0)	10 (8–13)	78.16	
CCU	7 (3.5)	16 (15–17)	155.79	
Gynaecological Ward	4 (2.0)	10.5 (2.25–18)	98	
General	4 (2.0)	17.5 (14.75–18.75)	174.38	
**Position**				
Staff nurse	185 (92.5)	12 (8.5–15)	99.08	
Head nurse	11 (5.5)	14 (5–15)	97.45	0.035^b^
Supervisor	4 (2.0)	17.5 (14.75–18.75)	174.38	
**Residency**				
Tulkarm	47 (23.5)	13 (10–16)	111.85	
Nablus	67 (33.5)	12 (9–15)	101.31	0.538^b^
Jenin	37 (18.5)	10 (8.5–14.5)	92.81	
Qalqelia	40 (20.0)	11.5 (7.25–15.75)	95.34	
Tubas	9 (4.5)	12 (8–14)	89.72	
**Years of working**				
Less than 5 years	49 (24.5)	12 (8–15.5)	100.11	
5 to less than10	71 (35.5)	12 (9–16)	104.74	0.710^b^
10 years or more	80 (40.0)	12.5 (8–15)	96.98	
**Educational status**				
Diploma	51 (25.5)	11 (7–15)	93.18	
BS	129 (64.5)	12 (9–15)	100.48	0.230^b^
MS	20 (10.0)	14 (9.25–15.75)	119.28	
**CPR Training**				
Yes	175 (87.5)	13 (9–15)	102.83	0.131^a^
No	25 (12.5)	9 (7–16.5)	84.18	

*ER* emergency room, *ICU* intensive care unit, *NICU* neonatal intensive care unit, *MW* medical word, *CCU* cardiac/coronary care unit, *BS* bachelor degree, *MS* master degree

^a^Statistical significance of differences calculated using the Mann–Whitney U-test

^b^Statistical significance of differences calculated using the Kruskal–Wallis test

Table 3 shows the knowledge score by sociodemographic variables score. A high knowledge score was associated with men (*p* = 0.001), and also shows a significant difference according to the department they worked, with a high knowledge score associated with the CCU, ICU, and general (*p* < 0.001); moreover, there was a significant difference between nurses according to the position and knowledge score. A high knowledge score was associated with being a supervisor (*p* = 0.035). No significant differences were observed between nurses according to age, marital status, hospital, residency, years of work, educational status, and CPR training.

### Description of the obstacles that nurses encountered

Table 4 describes the obstacles nurses had when giving the medications to patients that caused ME to occur. The results show that about half of the nurses (46.5%) agreed that an obstacle was that the names of many medications were similar, but 40% disagreed. More than half of the nurses (60%) agreed that the packaging was also an obstacle, while only (38%) agreed that mixing of resuscitation medications with other drugs was an obstacle. About half of the nurses agreed that the abbreviations used instead of writing the orders out completely and oral orders instead of written orders were not helpful. Confusing prescriptions and unclear dose calculations were also obstacles, but 42% disagreed that the pharmacy delivered incorrect doses and 31.5% agreed with this; 46.5% disagreed that the pharmacy did not label the medication correctly compared to 38% disagreed, and more than half (61%) agreed that pharmacists not staying all day was an obstacle (23% disagreed). 39% agreed that poor communication between physicians and nurses was an obstacle and 43% did not; only 26.5% disagreed that there was insufficient knowledge on resuscitation medications.

**Table 4:** Description of obstacles encountered by nurses during medication administration

Variable	Frequency (%)
**The similarity of medications name**	
Strongly agree	29 (14.5)
Agree	64 (32.0)
Neither agree or disagree	27 (13.5)
Disagree	65 (32.5)
Strongly disagree	15 (7.5)
**Different medications look-alike in the packaging**	
Strongly agree	33 (16.5)
Agree	89 (44.5)
Neither agree or disagree	20 (10.0)
Disagree	38 (19.0)
Strongly disagree	20 (10.0)
**Mixing of resuscitation medications with other drugs**	
Strongly agree	34 (17.0)
Agree	42 (21.0)
Neither agree or disagree	25 (12.5)
Disagree	68 (34.0)
Strongly disagree	31 (15.5)
**Use Abbreviations in place of writing the whole orders**	
Strongly agree	36 (18.0)
Agree	67 (33.5)
Neither agree or disagree	33 (16.5)
Disagree	52 (26.0)
Strongly disagree	12 (6.0)
**Verbal orders are used instead of written orders**	
Strongly agree	41 (20.5)
Agree	65 (32.5)
Neither agree or disagree	34 (17.0)
Disagree	40 (20.0)
Strongly disagree	20 (10.0)
**Confused prescription**	
agree Strongly	40 (20.0)
Agree	61 (30.5)
Neither agree or disagree	44 (22.0)
Disagree	41 (20.5)
Strongly disagree	14 (7.0)
**Unclear dose calculation**	
agree Strongly	32 (16.0)
Agree	64 (32.0)
Neither agree or disagree	37 (18.5)
Disagree	52 (26.0)
Strongly disagree	15 (7.5)
**Pharmacy delivers incorrect doses**	
Strongly agree	26 (13.0)
Agree	37 (18.5)
Neither agree or disagree	51 (25.5)
Disagree	63 (31.5)
Strongly disagree	23 (11.5)
**The pharmacy does not label the medication correctly**	
Strongly agree	27 (13.5)
Agree	49 (24.5)
Neither agree or disagree	31 (15.5)
Disagree	74 (37.0)
Strongly disagree	19 (9.5)
**Unavailability of pharmacists throughout the day**	
Strongly agree	52 (26.0)
Agree	70 (35.0)
Neither agree or disagree	32 (16.0)
Disagree	34 (17.0)
Strongly disagree	12 (6.0)
**Lack of communication between doctors and nurses**	
Strongly agree	27 (13.5)
Agree	51 (25.5)
Neither agree or disagree	36 (18.0)
Disagree	70 (35.0)
Strongly disagree	16 (8.0)
**Insufficient knowledge regarding resuscitation medications**	
Strongly agree	41 (20.5)
Agree	56 (28.0)
Neither agree or disagree	50 (25.0)
Disagree	40 (20.0)
Strongly disagree	13 (6.5)
**Perception of uncertain answers from other nurses**	
Strongly agree	34 (17.0)
Agree	73 (36.5)
Neither agree or disagree	41 (20.5)
Disagree	45 (22.5)
Strongly disagree	7 (3.5)
**Divergence of opinions between doctor and nurse**	
Strongly agree	41 (20.5)
Agree	68 (34.0)
Neither agree or disagree	35 (17.5)
Disagree	48 (24.0)
Strongly disagree	8 (4.0)
**No references for the use of resuscitation medications**	
Strongly agree	48 (24.0)
Agree	51 (25.5)
Neither agree or disagree	39 (19.5)
Disagree	52 (26.0)
Strongly disagree	10 (5.0)
**Embarrassment from asking colleagues about resuscitation drugs**	
Strongly agree	30 (15.0)
Agree	60 (30.0)
Neither agree or disagree	37 (18.5)
Disagree	57 (28.5)
Strongly disagree	16 (8.0)
**Interruption during drug administration to do other tasks at the same time**	
Strongly agree	40 (20.0)
Agree	57 (28.5)
Neither agree or disagree	36 (18.0)
Disagree	54 (27.0)
Strongly disagree	13 (6.5)
**General mess during resuscitation as many people are handling the same medication**	
Strongly agree	61 (30.5)
Agree	63 (31.5)
Neither agree or disagree	34 (17.0)
Disagree	36 (18.0)
Strongly disagree	6 (3.0)
**Shortage of resuscitation medications and need to borrow from other wards**	
Strongly agree	47 (23.5)
Agree	60 (30.0)
Neither agree or disagree	32 (16.0)
Disagree	48 (24.0)
Strongly disagree	13 (6.5)
**The nurse is unaware of a known allergy**	
Strongly agree	49 (24.5)
Agree	69 (34.5)
Neither agree or disagree	31 (15.5)
Disagree	42 (21.0)
Strongly disagree	9 (4.5)

Table 4 also shows that more than half (53.5%) of nurses took unconfirmed information from colleagues, moreover, 54.5% of them agreed that there was a disagreement of opinions between doctors and nurses. 49.5% of nurses said that there were no references to refer to for resuscitation medications, and only 31% disagreed. 45% of nurses were embarrassed to ask questions about resuscitation medications and 36.5% did not. It was acknowledged that interruptions during drug administration procedures among nurses (e.g., being asked to handle other tasks) were an obstacle for about half of them (48.5%), but not for 33.5%. Only 21% did not consider that the general chaotic conditions in the CPR procedure (such as many people handling a single drug) was an obstacle, and more than half of nurses considered that deficiency and inaccessibility to resuscitation medications and the ignorance of patient allergies were obstacles.

### Classify nurses according to knowledge level and training need

Table 5 describes that 60% of nurses see that they have ‘sufficient’ or “relatively sufficient” knowledge levels about resuscitation medications and only 19% see that they have “insufficient knowledge”; most of them (70.5%) said that they needed further training about resuscitation medications.

**Table 5:** Self-evaluation of knowledge level and training needs for resuscitation medications

Variable	Frequency (%)
**In your opinion, your knowledge level about resuscitation medications is;**	
Sufficient	38 (19.0)
Relatively sufficient	82 (41.0)
Fair	42 (21.0)
Insufficient	32 (16.0)
Extremely insufficient	6 (3.0)
**In your opinion, do you need training about resuscitation medications:**	
Need	141 (70.5)
No comment	32 (16.0)
No need	27 (13.5)

### Causes of medication administration errors not reported

Table 6 shows why medication administration errors are not reported. More than half of nurses (58%) agreed that the differences between the nurses’ and hospital definition of a “ME” was a reason not to not report an error, however, 48% of them did not consider that not recognising errors occurred as a reason for this (15% neutral). 41.5% of nurses did not consider that filling out an incident report for an EM takes a long time (21% neutral), while 42.5% considered that contacting the physician about an error takes a long time and was a reason not to report an error (18.5% neutral). More than half of the nurses (53.5%) agreed that ME are defined vaguely, while half of them (49.5%) disagreed that nurses think that the mistake was not important to report (16.5% neutral).

**Table 6:** Causes of medication administration errors not reporting

Variable	Frequency (%)
**There is no compatibility in the definition of a medication error between hospitals and nurses**	
Strongly agree	29 (14.5)
Agree	87 (43.5)
Neither agree or disagree	37 (18.5)
Disagree	42 (21.0)
Strongly disagree	5 (2.5)
**Nurses aren't aware of the error happening**	
Strongly agree	23 (11.5)
Agree	51 (25.5)
Neither agree or disagree	30 (15.0)
Disagree	66 (33.0)
Strongly disagree	30 (15.0)
**Filling out the incident report form takes a lot of time**	
Strongly agree	29 (14.5)
Agree	46 (23.0)
Neither agree or disagree	42 (21.0)
Disagree	70 (35.0)
Strongly disagree	13 (6.5)
**It takes a lot of time to contact a doctor about a medication error**	
Strongly agree	26 (13.0)
Agree	59 (29.5)
Neither agree or disagree	37 (18.5)
Disagree	67 (33.5)
Strongly disagree	11 (5.5)
**The definition of a medical error is not obvious**	
Strongly agree	30 (15.0)
Agree	77 (38.5)
Neither agree or disagree	35 (17.5)
Disagree	49 (24.5)
Strongly disagree	9 (4.5)
**Nurses believe a medical error is insignificant enough to document**	
Strongly agree	24 (12.0)
Agree	44 (22.0)
Neither agree or disagree	33 (16.5)
Disagree	73 (36.5)
Strongly disagree	26 (13.0)
**Nurses believe that their colleagues will think they are incompetent when making a medical error**	
Strongly agree	40 (20.0)
Agree	70 (35.0)
Neither agree or disagree	29 (14.5)
Disagree	44 (22.0)
Strongly disagree	17 (8.5)
**Patients or their families might have developed a negative attitude toward the nurses, or take legal action if they report a medication error**	
Strongly agree	50 (25.0)
Agree	85 (42.5)
Neither agree or disagree	24 (12.0)
Disagree	33 (16.5)
Strongly disagree	8 (4.0)
**Nurses fear that the doctor will blame them for medical errors**	
Strongly agree	33 (16.5)
Agree	59 (29.5)
Neither agree or disagree	35 (17.5)
Disagree	52 (26.0)
Strongly disagree	21 (10.5)
**Fear of consequences when reporting a medical error**	
Strongly agree	53 (26.5)
Agree	70 (35.0)
Neither agree or disagree	25 (12.5)
Disagree	39 (19.5)
Strongly disagree	13 (6.5)
**The nursing management response does not match the severity of the error**	
Strongly agree	36 (18.0)
Agree	73 (36.5)
Neither agree or disagree	47 (23.5)
Disagree	38 (19.0)
Strongly disagree	6 (3.0)
**Fear of blame if something bad happened to the patient because of a medical error**	
Strongly agree	59 (29.5)
Agree	84 (42.0)
Neither agree or disagree	29 (14.5)
Disagree	18 (9.0)
Strongly disagree	10 (5.0)
**Lack of appreciation when administering medication in a healthy way**	
Strongly agree	42 (21.0)
Agree	76 (38.0)
Neither agree or disagree	41 (20.5)
Disagree	34 (17.0)
Strongly disagree	7 (3.5)
**The reliance on medical errors as a measure of the quality of the nursing care provided**	
Strongly agree	47 (23.5)
Agree	78 (39.0)
Neither agree or disagree	35 (17.5)
Disagree	30 (15.0)
Strongly disagree	10 (5.0)
**Management concentrate on the individual rather than the system as the cause of the medical error**	
Strongly agree	59 (29.5)
Agree	84 (42.0)
Neither agree or disagree	25 (12.5)
Disagree	26 (13.0)
Strongly disagree	6 (3.0)

55% of nurses thought that other nurses would think they are unqualified if they make ME and so did not report it, and 67.5% were afraid that patients or their families might develop a negative attitude toward them, or take legal action against them, and so they did not report ME (12% neutral). 46% were worried that the physician would reprimand them for ME (17.5% neutral), and more than half (61.5%) of the nurses panicked about adverse consequences of reporting ME (12.5% neutral); 54% of them agreed that the nursing administration did not take the proper action that matched the severity of the errors (23.5% neutral) and most of the nurses (71.5%) said that they could be blamed if something happened to the patient as a consequence of ME and so did not report it. 59% said that they did not see encouragement for correctly administering medication (20.5% neutral), but a high value was attributed to an EM as an indication of medical care provided by nurses, therefore, 62.5% of nurses did not report errors; most nurses (71.5%) noticed that nursing administrators focused on the person rather than the system when a ME occurred.

### Suggestions for decreasing ME

Table 7 shows that most nurses strongly agreed on the solutions I suggested to them: 1) better arrangement of medications by names, labels, and packages can increase correct and safe use of healthcare providers (71.5%); 2) create a continuous learning and training program for nursing staff (71%); 3) prepare a trained CPR team is necessary for professional resuscitation action (65%) called “code blue”; 4) provide better access to reference information about drugs (69%), and 5) provide a clinical pharmacist in the departments as a reference for medicines to help nurses (66.5%).

**Table 7:** Suggestion for decrease medication errors

Variable	Frequency (%)
**Good arrangement of medications by names, labels, and packages can increase correct and safe use by healthcare providers**	
Strongly agree	143 (71.5)
Agree	46 (23.0)
Neither agree or disagree	6 (3.0)
Disagree	2 (1.0)
Strongly disagree	3 (1.5)
**Make continuous learning and training to nurses’ staff**	
Strongly agree	142 (71.0)
Agree	44 (22.0)
Neither agree or disagree	8 (4.0)
Disagree	5 (2.5)
Strongly disagree	1 (0.5)
**Preparing a trained CPR team is necessary for professional resuscitation action**	
strongly agree	130 (65.0)
Agree	55 (27.5)
Neither agree or disagree	11 (5.5)
Disagree	3 (1.5)
Strongly disagree	1 (0.5)
**Provide a more effective source or reference for information about the drug**	
Strongly agree	138 (69.0)
Agree	47 (23.5)
Neither agree or disagree	9 (4.5)
Disagree	5 (2.5)
Strongly disagree	1 (0.5)
**Providing clinical pharmacists in the departments as a reference for medicines to help nurses**	
Strongly agree	133 (66.5)
Agree	44 (22.0)
Neither agree or disagree	14 (7.0)
Disagree	7 (3.5)
Strongly disagree	2 (1.0)

*CPR* Cardiopulmonary resuscitation

In addition, some of the nurses suggested other solutions, such as increasing the number of nurses on staff to decrease the general workload, establishing an electronic medical library within reach of nursing hands, and giving value to the nursing role when dealing with them and not treating them just as a tool to execute orders.

## Discussion

This study is one of the first in Palestine that has been performed to determine factors that affect nurses' knowledge about resuscitation medication, discuss the obstacles they encountered during medication administration, explain the causes behind not reporting ME and suggest solutions to decrease ME.

Insufficient knowledge among nurses is considered one of the most important reasons for medication administration errors [3, 4, 42].

In our study, the correct answer rate regarding high-alert medications was only 58.6%, which was quite low compared to a similar study done in Taiwan 70.5% [32], and 60.9%, 56.5%, 75.8% in studies done in Palestine and Taiwan, respectively [38, 39, 42], also proves that nurses are lacking information about resuscitation medications and high-alert medications. The question about giving 15% KCl as an IV push in emergency cases such as ventricular fibrillation achieved the correct answer rate of 87.5%, which showed good knowledge of how to avoid ME which can cause cardiac arrest and death; It was a higher rate compared to a study done in Palestine on high alert medications which was 76.8% [42], but lower than a similar study conducted in Taiwan (95.2%) [32], and the same result as Lu et al. [38]. 68.5% of nurses gave a correct answer to not giving amiodarone by the trachea to increase the effects, which is considered high compared to the same study done in Taiwan, which only achieved 42.0% [32], but still low, because there is no absorption for amiodarone through the trachea [43]. On the contrary, 63.5% of nurses still thought that atropine could be used in pulseless electrical activity treatment, which was the lowest correct answer rate, while only 29.8% of nurses answered incorrectly / do not know about this question [32] which indicates low knowledge. The next lowest correct answer rate was 44.5%, representing that more than half of the nurses who did not know that body weight is used in an epinephrine dose calculation for resuscitation of a child, which is a comparable result with a study conducted in Taiwan [32]. Calcium chloride (CaCl_2_)injection should be administered gradually in a large vein, but 53.5% of nurses did not recognize this error; the same result was found by Zyoud et al. [42], while only 39.2%, 49% of nurses incorrectly answered in studies done in Taiwan [32, 38]. 32% of nurses incorrectly answered about the inability to switch between 10% Ca gluconate and 10% CaCl2, which is the same result in a study conducted in Palestine [42], but a lower correct rate than studies in Taiwan [32, 38].

To increase nurses’ pharmacological knowledge of resuscitation medication and increase patient safety, the common obstacles that nurses face and the ones that lead them to make errors should be known. In our study, we found that the major obstacle nurses faced when administering resuscitation medication was a chaotic environment during CPR, for example, with many people handling a single drug (62%). This was the third of 12 obstacles that nurses faced in a study completed by Chen et al. [32], and Ornato et al. also mentioned that too many individuals present in the resuscitation room, as well as poor teamwork and leadership, could result in errors in the resuscitation system [44]. Furthermore, the inaccessibility of pharmacists for the whole day (61%) and the similarity of different medications packaging (61%) were further obstacles.

Several studies found that a deficiency in pharmacological knowledge is the major reason for ME. For example, inadequate knowledge was the main obstacle that nurses faced in Taiwan (75.4%) [39], and 28.2% of nurses considered it an obstacle [32]. Furthermore, in our research, 48.5% of nurses considered having inadequate knowledge as a major obstacle, while another study considered interruptions during drug administration as the main obstacle 62.8% [32], while 48.5% of nurses in our study agreed with that, another studied poor communication between nurses and physicians was an important obstacle [11, 42]. In our study, 39% of nurses considered it essential to establish more trained and cooperative CPR teams for professional resuscitation action.

38% of nurses believed they had sufficient knowledge levels about resuscitation medications, while only 8% of nurses in Taiwan thought that [32], and only 3.6% and 23.6% of nurses believed themselves to have adequate knowledge on high-alert medications in Taiwan and Palestine, respectively [39, 42]. Most nurses acknowledged that they needed training on resuscitation medications as well as other studies [32, 39, 42].

In our study, we found that there was no compatibility in the definition of ME between nurses and hospitals (43.5%), which was the main reason for not reporting medication administration. The other most important causes were the negative attitudes that patients and their families towards the nurse or that they may litigate against the nurse if an EM is reported (42.5%). If something happens to the patient, nurses thought that they would be blamed as a consequence of ME (42%), and that nursing management focused on the person (rather than the system) when ME occurs (42%) [45, 46]. The study by Mansouri et al. mentioned that the ME were not documented for three reasons: fear of consequences after reporting an error, procedural obstacles and management problems [47], and in our study, fear of adverse consequences was an important barrier to reporting incidents.

To improve ME reporting, we have to create an encouraging and supportive environment with no blame and dishonor when genuine mistakes are made. Teaching nurses how and what to report incidents and giving rewards to encourage incident reporting would help. Increasing the provision of corrective actions regarding incident reports, such as training health providers to recognise medical incidents, and the existence of a reporting system, would be beneficial [45].

We suggested solutions to increase patient safety, and the nurses agreed with them. These were; an improved arrangement of drug names, labels, and packages can increase proper use of medications by healthcare providers and improve patient safety; create a continuous learning and training program for nurses and staff [6, 42, 48], prepare more trained and cooperative CPR teams to increase the efficiency of resuscitation actions [44]; provide a more effective source of reference for information about drugs, such as clinical pharmacy service, as it positively impacts the quality of care [49].

The nurses were found to lack sufficient information about the drugs they are giving. A positive direct effect was shown between the knowledge score and the male participants (*p* = 0.001). These findings are comparable with a study conducted by Zyoud et al. on high-alert drugs [42] and nurses working in CCU, ICU, and general wards (*p* < 0.001). In Taiwan, the same result was found that nurses who worked in the ER or ICU departments had more knowledge than those who worked in the EMS and obstetric-paediatric wards [32]. In addition, on the medical and surgical wards, medical errors occurred more frequently, according to Sheu et al. [50].

Our study observed that there was a significant difference according to the position of the nurses, with supervisor nurses having a higher score compared to staff and principal nurses (*p* = 0.035); Zyoud et al. also considered the position as a factor affecting the knowledge score and found head nurses had the highest score [42].

There was no significant relationship between the knowledge score and age, marital status, hospital, residency, years of work, education level, and CPR training. However, a positive relationship between years of work and the knowledge score had been found in several studies [32, 38, 39], educational level and the knowledge score [42], and also between the CPR training and the knowledge score [32].

### Strengths and limitations

This study was one of the first to evaluate the knowledge and understanding of the obstacles they face when giving resuscitation medications to patients and the low reporting of ME in Palestine. However, our study had some limitations. First, this is a cross-sectional study and it is therefore difficult to prove causal relationships between the scales and their associated factors. Second, because the information was collected through face-to-face interviews, the interviewer’s bias can influence the results. Third, samples were collected only from the north area of the West Bank, which could be considered a limiting factor. Finally, we included all working nurses at the time of the study period and did not consider the status of emotional or physical health situations during the selection process.

### Implications for research/practice

This study is the first in the field of nursing in Palestine to concern nurses' knowledge and obstacles when administering resuscitation medications. Establish a database for future studies in different professions in the medical field, and the results of this study help health care providers and health policymakers create mechanisms to decrease ME and establish clear protocols to increase patient safety as possible and improve health care in Palestinian hospitals. Nurses indicated that many factors and obstacles are the reasons for resuscitation medication administration errors, such as chaotic environment and the unavailability of pharmacists for a whole day. These obstacles should be considered to improve practice. Therefore, an evaluation of nurses' knowledge is necessary to measure the degree of lack of information about resuscitation medications and to recognise the obstacles they face during medication administration. This will enable future activities and strategies to significantly and decrease deaths.

## Conclusions

In conclusion, in our study, the correct answer rate was only 58.6%, which indicates poor knowledge among nurses, which is an obstacle that causes ME. Furthermore, this study has shown that a higher knowledge score was found in male nurses and nurses working in the CCU, ICU, and general departments, but no differences were observed between nurses with regard to age, marital status, hospital, residency, years of work, educational status, and CPR training.

This study identifies the causes of drug administration-related errors by nurses during cardiac resuscitation and offers solutions. It can be helpful when others perform similar studies in their own health care systems and geographical localities. According to our results, a chaotic condition in CPR conditions were the most common obstacle that nurses faced in administering resuscitation medication at a rate of 62%. Furthermore, there is no common agreement; also would like to have extra training to improve and update their pharmacology information. According to the results and conclusions of this study, pharmacists must better organize medications so that names, labels, and packages are clearer to find and use. Continuous learning and training for nurses staff, better preparation and organized training for CPR teams to carry out resuscitation actions in a coordinated and professional way as a code blue team; providing a more effective source of reference material for information about drugs for nurses, and providing a clinical pharmacist in all departments as reference help nurses.

This study gives a great deal of insight into the clinical context, especially for nurses’ practice. Therefore, comprehensive actions that can reduce nurses’ make fewer errors should be made following the study findings. First, hospitals should increase the number of nursing staff to reduce their workload on them. Second, establishing an electronic medical library within reach of nursing hands could give nurses valuable tools to execute orders. Lastly, organising a calm and disciplined CPR environment can improve communication between nurses and physicians to reduce ME.

## Data Availability

The data sets used and/or analyzed during the current study are available from the corresponding authors on reasonable request.

## Abbreviations

Amp: Ampoule
BS: Bachelor's Degree
Ca: Calcium
CaCl_2_: Calcium Chloride
CCU: Cardiac / Coronary Care Unit
CPR: Cardiopulmonary Resuscitation
ER: Emergency Room
ICU: Intensive Care Unit
IRB: Institutional Review Boards
IV: Intravenous
KCl: Potassium Chloride
ME: Medication Error
MS: Master’s Degree
MW: Medical Word
NaHCo_3_: Sodium Bicarbonate
NICU: Neonatal Intensive Care Unit
NTI: Narrow Therapeutic Index
SD: Standard Deviation
SPSS: Statistical Package for Social Sciences
